# Reactivating p53 functions by suppressing its novel inhibitor iASPP: a potential therapeutic opportunity in p53 wild-type tumors

**DOI:** 10.18632/oncotarget.4847

**Published:** 2015-07-13

**Authors:** Peixin Dong, Kei Ihira, Junichi Hamada, Hidemichi Watari, Takahiro Yamada, Masayoshi Hosaka, Sharon J.B. Hanley, Masataka Kudo, Noriaki Sakuragi

**Affiliations:** ^1^ Department of Women's Health Educational System, Hokkaido University School of Medicine, Hokkaido University, Sapporo, Japan; ^2^ Department of Gynecology, Hokkaido University School of Medicine, Hokkaido University, Sapporo, Japan; ^3^ Department of Stem Cell Biology, Hokkaido University Graduate School of Medicine, Kita-Ku, Sapporo, Japan

**Keywords:** review, reactivation of p53, iASPP, microRNA, invasion

## Abstract

Although mutational inactivation of p53 is found in 50% of all human tumors, a subset of tumors display defective p53 function, but retain wild-type (WT) p53. Here, direct and indirect mechanisms leading to the loss of WT p53 activities are discussed. We summarize the oncogenic roles of iASPP, an inhibitor of WT p53, in promoting proliferation, invasion, drug or radiation-resistance and metastasis. From the therapeutic view, we highlight promising perspectives of microRNA-124, peptide and small molecules that reduce or block iASPP for the treatment of cancer. High iASPP expression enhances proliferation, aggressive behavior, the resistance to radiation/chemotherapy and correlates with poor prognosis in a range of human tumors. Overexpression of iASPP accelerates tumorigenesis and invasion through p53-dependent and p53-independent mechanisms. MicroRNA-124 directly targets *iASPP* and represses the growth and invasiveness of cancer cells. The disruption of iASPP-p53 interaction by a p53-derived peptide A34 restores p53 function in cancer cells. The inhibition of iASPP phosphorylation with small molecules induces p53-dependent apoptosis and growth suppression. The mechanisms underlying aberrant expression of iASPP in human tumors should be further investigated. Reactivating WT p53 functions by targeting its novel inhibitor iASPP holds promise for potential therapeutic interventions in the treatment of WT p53-containing tumors.

## INTRODUCTION

The initiation and progression of cancer is a multistep process including self-renewal, aberrant cell cycle, defective apoptosis, the induction of the epithelial to mesenchymal transition (EMT), enhanced mobility, invasion and angiogenesis, and the remodeling of the tumor microenvironment [1]. In addition to numerous genetic alterations, such as inactivation of a tumor suppressor and activation of an oncogene, human tumor cells harbor global epigenetic abnormalities, such as DNA methylation, histone modifications, nucleosome positioning and non-coding RNAs [2]. Genetic and epigenetic changes interact with each other to enable cancer progression [3].

MicroRNAs (miRNAs) are characterized as endogenous, small size, non-coding RNA molecules that post-transcriptionally control the translation and stability of mRNAs [4]. MiRNAs are predicted to regulate the expression of approximately 60% of human genes [5]. A single miRNA can bind to multiple mRNAs via perfect or partial base-pairing with the 3′-untranslated region (UTR) of the target mRNAs [5], resulting in profound effects on gene expression and cellular functions. MiRNAs modulate tumor cell proliferation, invasion and EMT/cancer stem cell (CSC) properties by targeting multiple downstream genes or signaling pathways [6, 7, 8].

**Figure 1:**
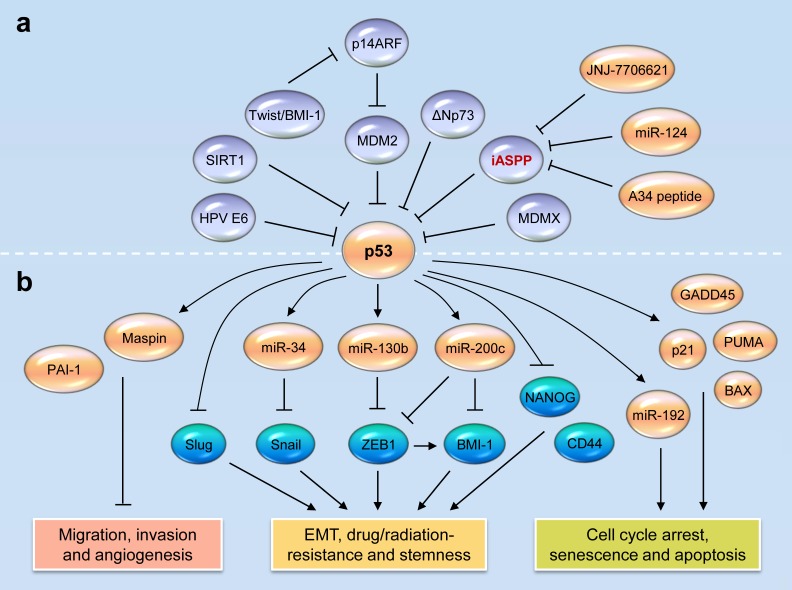
Reactivating wild-type p53 functions in tumors by targeting its novel inhibitor iASPP **a.** Direct and indirect mechanisms of p53 inactivation in human tumors. iASPP acts as a key p53 inhibitor. MiR-124 negatively regulates the expression of iASPP in human tumors. The disruption of iASPP-p53 interaction by a p53-derived peptide A34 restores p53 function in cancer cells. The inhibition of iASPP phosphorylation with small molecule JNJ-7706621 induces p53-dependent apoptosis and growth suppression. **b.** Wild-type p53 represses cancer initiation, progression and metastasis by regulating downstream genes and microRNAs (miR-34, miR-130b, miR-192 and miR-200c)-target gene networks.

The p53 family members (p53, p63 and p73) play a pivotal role in the regulation of many critical biological processes including cell death, proliferation, cell cycle control and tumorigenesis [9, 10, 11, 12]. When DNA damage is sensed, the apical kinases ATM and ATR checkpoint pathways are triggered, and in turn activates and stabilizes wild-type (WT) p53 via the phosphorylation of CHK2/CHK1, or degradation of MDM2 [13]. Once activated by various cellular stresses, WT p53 accumulates in the nucleus and works as a transcriptional factor to either transactivate or transrepress downstream genes or miRNAs, leading to the induction of growth arrest, cellular senescence and apoptosis, the inhibition of angiogenesis and metastasis in tumors [9, 14, 15, 16] (Figure 1). In addition to these anticancer effects, a new role of p53 has emerged, regulation of EMT and CSC features [9, 17, 18]. During the metastatic cascade, tumor cells usually activate the EMT, a dynamic cellular process thought to be critical step of metastasis by promoting the acquisition of migratory and invasive capabilities and gain of CSC-like phenotypes, such as the resistance to radiation or chemotherapy [1]. Several transcription factors including Snail, Slug, Twist and ZEB-1/2 induce EMT, by downregulating the epithelial markers such as E-cadherin and by upregulating the mesenchymal markers such as vimentin [1]. By targeting *Slug* and *Snail*, p53 negatively regulates EMT and suppresses cancer cell invasiveness [19, 20]. Emerging evidence has demonstrated that WT p53 can also indirectly silence EMT-inducing transcription factors though the transcriptional regulation of some miRNAs, such as miR-34, miR-130b, miR-145, miR-192, miR-215 and miR-200c [21, 22, 23, 24, 25, 26, 27]. For example, by binding to the promoter region of *miR-130b*, p53 transactivates this miRNA to reduce the levels of ZEB1 (a direct target gene of miR-130b), and thereby attenuates EMT and invasiveness in endometrial cancer cells [22]. These studies support the idea that the disruption of p53 tumor suppressor-regulated pathways contributes to progression and worse clinical outcome of human tumors [28].

In this review, we provide a brief overview of the mechanisms by which the functions of WT p53 are inactivated and will focus on the oncogenic roles of iASPP, a negative regulator of WT p53, in human tumors. We will discuss current approaches that aim to restore p53 functions by targeting iASPP, including miR-124, a peptide derived from p53 and other small molecules.

## DIRECT AND INDIRECT MECHANISMS LEADING TO THE LOSS OF WT P53 ACTIVITIES IN HUMAN TUMORS

The loss of p53 function in human cancers occurs either by direct or indirect mechanisms (Figure 1a). At least half of all tumors lose WT p53 function as a result of mutations [29], however a subset of tumors, such as cervical cancer, chronic lymphocytic leukaemia, acute lymphoblastic leukaemia, acute myeloblastic leukaemia, myeloma, neuroblastoma, melanoma, mantle cell lymphoma and sarcoma, retains WT p53, but its activity can be attenuated [30, 31]. MDM2 is the best-known inhibitor of p53. MDM2 limits p53 activity by binding to and blocking the N-terminal trans-activation domain of p53, or by working as an E3 ubiquitin ligase to promote p53 degradation [32, 33]. MDM2 is directly inhibited by the tumor suppressor p14ARF, loss of which also leads to reduced p53 [34]. MDMX, a homolog of MDM2, can interact with p53 and repress its activities [35]. Moreover, the EMT inducers Twist and BMI-1 were shown to mediate the inhibition of p53 pathway via suppressing p14ARF [36, 37]. The E6 oncoproteins of high-risk human papillomaviruses (HPV) bind p53 and are capable of inducing its degradation [38]. Human *TP73* gene produces an NH2 terminally truncated isoform, ΔNp73 that lacks the transactivation domain and function as a dominant-negative inhibitor of WT p53 [39]. Other mechanisms responsible for loss of WT p53 functions in human tumors include the lack of p53 nuclear retention [40] and the deacetylation of p53 by Sirtuin 1 (SIRT1) [41]. Thus, the reactivation of endogenous p53, either by reducing or blocking its negative regulators, would benefit cancer patients with WT p53 tumors [42].

## THE ONCOGENIC ROLES OF IASPP IN HUMAN TUMORS

To date, intensive efforts have been made to restore WT p53 activity as an anticancer therapeutic approach [42]. Reducing the levels of p53 suppressors or interfering with the direct physical association between p53 suppressors and p53, has shown effectiveness for the restoration of p53 function. For example, Withaferin A (WA), a small-molecule natural compound, downregulates the expression of HPV E6 oncoprotein and helps to restore p53-dependent apoptosis in cervical cancer cells [43]. Given that the inhibition of p53 by MDM2 protein relies on their direct binding with p53, small-molecule MDM2 inhibitors, such as Nutlin-3 that disrupts this interaction, have been utilized to induce WT p53-mediated cell-cycle arrest and apoptosis in various types of tumor cells [42]. However, tumor cells may develop the resistance to Nutlin-3 owing to the fact that this MDM2 inhibitor does not efficiently target the MDMX-p53 interaction or fails to induce MDMX degradation [44]. More importantly, the continuous Nutlin-3 exposure can result in the acquisition of somatic mutations in p53 and select for p53-mutated cells in solid tumors [45, 46]. Therefore, additional attempts to achieve p53 activation through targeting other p53 inhibitors are required for effective tumor regression.

The ASPP family consisting of three proteins (ASPP1, ASPP2 and iASPP) interacts with and modulates the functions of WT p53 [47]. ASPP1/2 enhances p53-dependent cell death, whereas iASPP inhibits the apoptotic transactivation potential of p53 by direct interaction [47]. Here, we review the oncogenic functions of iASPP in tumors and discuss promising therapeutic perspectives of miRNA, peptide and small molecules that reduce or block iASPP for the treatment of cancer.

Human iASPP, which is encoded by *PPP1R13L* located on 19q13.2-3 [48], has two isoforms (407 and 828 amino acids-aa) [49, 50]. The shorter form of iASPP (407 aa) is a nuclear protein, and the longer form of iASPP (828 aa) is located in both the nucleus and cytoplasm [48, 49, 50]. iASPP was considered as an oncogene that not only inhibits the transcriptional activity of p53 on promoters of downstream genes (Figure 1a), but also promotes carcinogenesis through p53-independent mechanisms, mainly by inhibiting the apoptotic activity of p63 and p73 [51]. Of note, in normal cells, iASPP can actually induce apoptosis via inhibition of nuclear factor-κB (NF-κB) [52].

iASPP is overexpressed in diverse human tumors, including colorectal cancer [53], acute leukemia [54], endometrioid endometrial cancer [55], lung cancer [56], glioblastoma [57], head and neck squamous cell carcinoma [58], prostate cancer [59], hepatocellular carcinoma [60, 61, 62], oral squamous cell carcinoma [63, 64], cervical cancer [65] and ovarian cancer [66]. Elevated expression of iASPP correlates with poor survival in head and neck cancer [58], oral squamous cell carcinoma [64], cervical cancer [65] and ovarian cancer [66]. Increased iASPP expression is associated with tumor grade, invasion and lymph node metastasis in endometrial cancer [55]. Furthermore, siRNA- or shRNA-mediated iASPP silencing reduces *in vitro* proliferation in human cancer cells [56, 59, 60, 61, 62, 63, 67, 68, 69]. The overexpression of iASPP in primary mouse embryonic fibroblasts promotes p53 degradation and strongly stimulates cell migration and metastasis [70]. These data suggest that iASPP contributes to the progression and metastasis of human tumors.

Human cancers are composed of heterogeneous cell populations. Genetic and phenotypic variation exists not only between different tumor types or between patients with the same tumor type (inter-tumor heterogeneity), but also within a single tumor (intra-tumor heterogeneity) [71]. This intra-tumor heterogeneity produces distinct subclones that show diverse malignant properties [72.]. It is thought that within a tumor, only a small population of tumor cells (CSC) is capable of self-renewal, multipotency, *in vivo* tumorigenicity and forming metastasis [73, 74]. This CSC model has been used to explain tumor heterogeneity. Accumulating evidence has linked p53 loss to stem-like phenotypes in cancers [9]. Importantly, knockdown of iASPP with siRNA impaired *in vivo* tumorigenesis of prostate cancer cells [59]. These data support the possible role of iASPP in promoting CSC properties, at least though inhibiting p53 activity.

On the other hand, the concept of CSC plasticity (the model of dynamic stemness) has been also proposed [75], in which CSC and non-CSC states may not be definitive, and cancer cells have the dynamic and transient ability of shifting from a non-CSC state to a CSC state as a consequence of EMT induction or microenvironmental stimuli [75]. The plasticity of CSC may at least partially help explain the tumor heterogeneity observed in tumors, and is consistent with those findings that tumor cell undergoing EMT can acquire stem cell-like properties [1].

Despite some results showing that the activation of EMT program suppresses the tumor-initiating properties of CSCs [76, 77], numerous studies have provided a strong link between EMT activation and the emergence of a CSC-like phenotype in human cancers [78]. In endometrial cancer, oncogenes such as *p53* gain-of-function mutations [21], *KLF17* [79], *EZH2*, *MCL-1* and *FOS* [8] promote EMT-associated invasiveness and enhance CSC properties including self-renewal capacity and chemoresistance, whereas miR-101, miR-106b, miR-130b and miR-194 [7, 8, 22, 80] serve as EMT suppressors and attenuate CSC features. Of significance, CSC cells enriched with CD29 and CD44 markers exhibit molecular characteristics, consistent with EMT [81]. These data collectively suggest a close correlation between EMT induction and CSC characteristics.

The increased iASPP expression is correlated with the resistance to radiation or chemotherapy in cervical cancer [65]. The overexpression of iASPP in ovarian cancer cells confers resistance to paclitaxel [66]. Consistent with these observations, enhanced iASPP expression can render cells resistant to ultraviolet radiation and cisplatin-induced apoptosis in human tumors expressing WT p53 [82]. Given the anti-oncogenic effects of WT p53 on EMT and CSC phenotypes, we speculate that iASPP might have a regulatory role in enhancing EMT and CSC-related phenotypes, such as resistance to chemotherapy and radiation, by inhibiting WT p53 function [83]. More studies are clearly needed to address these possibilities.

## IASPP, A TARGET FOR ANTITUMOR THERAPEUTICS

The therapeutic targeting of iASPP might include targeting iASPP itself as well as modulating its upstream regulators or downstream effectors (Figure 1a). The Wnt/beta-catenin signaling seems to be an upstream regulator of iASPP in gastric cancer, because the attenuation of beta-catenin by shRNA resulted in apparent apoptosis and downregulated iASPP [84]. MiR-124 directly targets *iASPP* and represses the growth and invasiveness in a variety of cancer cells, including colorectal cancer, glioblastoma and prostate cancer [85, 86, 87]. In addition, A34 (a small peptide derived from p53 linker) binds directly to iASPP and competitively inhibits the iASPP-p53 interaction in human tumor cells [88], causing WT p53-mediated transcriptional activation of *Bax* and *PUMA* and tumor cell apoptosis *in vitro* and *in vivo* [88]. Furthermore, a small-molecule inhibitor of cyclin B/CDK1, JNJ-7706621 that inhibits iASPP phosphorylation and prevents the nuclear entry of iASPP, can induce WT p53-dependent apoptosis and growth suppression in melanoma cells [89]. The identification of miRNAs and molecules that control the levels of iASPP and a high-throughput screening to search for small-molecule compounds that release p53 from iASPP, would lead to the development of therapies against iASPP for the treatment of human tumors through activation of WT p53.

## CONCLUSIONS

In addition to affecting tumor cell proliferation and survival, WT p53 can repress cancer initiation, progression and metastasis by regulating downstream genes and miRNAs-target gene networks. A more complete understanding of genetic and epigenetic mechanisms underlying aberrant expression of WT p53 inhibitor iASPP in human tumors, and detailed studies on iASPP function in various aspects of tumor progression, including EMT, stemness and metastasis, can provide a basis for new potential therapeutic applications in WT p53-containing tumors.
